# A robotic DNA purification protocol and real-time PCR for the detection of *Enterobacter sakazakii *in powdered infant formulae

**DOI:** 10.1186/1471-2180-6-100

**Published:** 2006-12-13

**Authors:** Sylviane Derzelle, Françoise Dilasser

**Affiliations:** 1 Laboratoire d'Etudes et de Recherches sur la Qualité des Aliments et des Procédés agro-alimentaires, Agence française de sécurité sanitaire des aliments (AFSSA), 23 Avenue du Général de Gaulle, 94706 Maisons Alfort cedex, France

## Abstract

**Background:**

*Enterobacter sakazakii *is the causative agent of rare but severe food-borne infections associated with meningitis, necrotizing enterocolitis and sepsis in infants. Rehydrated powdered infant formulae have been implicated as the source of infection in several outbreaks and sporadic cases. In this work, a real time fluorescence resonance energy transfer PCR assay incorporating an internal amplification control (IAC) was developed for the specific detection of *E. sakazakii *in foods. Performance of the assay, coupled to an automated DNA extraction system and the *E. sakazakii *ISO-IDF (TS 22964/RM 210) enrichment procedure, was evaluated on infant formulae and samples from production environment.

**Results:**

The real-time PCR assay had 100% specificity as assessed using 35 *E. sakazakii *and 184 non-*E. sakazakii *strains. According to the *E. sakazakii *strains tested, the detection limits ranged from 5 to 25 genomic copies. Assays on pure cultures (including real-time PCR and DNA extraction) gave a sensitivity of about 10^2 ^to 10^3 ^CFU/ml. Out of 41 naturally contaminated infant formulae and environmental samples analysed for the presence of *E. sakazakii*, 23 were positive by real-time PCR and 22 by the conventional culture method, giving 97.5% concordance with the ISO-IDF reference method.

**Conclusion:**

This method, combining specific real-time PCR, automated DNA extraction and ISO-IDF standard enrichments, provides a useful tool for rapid screening of *E. sakazakii *in food and environmental matrices.

## Background

*Enterobacter sakazakii*, previously known as yellow-pigmented *E. cloacae *[1], has been identified as the causative agent of rare but often severe invasive infections in infants. Neonates and infants less than 2 months of age appear to be groups at particular risk [2,3]. Meningitis is the most frequently reported clinical symptom in neonatal *E. sakazakii *infections [2,4-7]. Infants in whom meningitis developed were generally < 1 week of age at the onset of infection and had near-term gestational age and birthweight [3]. They frequently develop complications including seizures and brain abscesses, resulting in high mortality (over 40%) and morbidity. Septicaemia or necrotizing enterocolitis are also associated with *E. sakazakii*-related infections [2,4-7]. Compared with infants with meningitis, infants in whom bacteremia alone developed tended to be born very prematurely and have extremely low birthweight (<1,000 g). They had generally surpassed the neonatal period at the onset of their disease [3]. Mortality among these cases is lower (about 10%) [2,4-7].

*E. sakazakii *is emerging as a hazard in powdered infant formulae (PIF). In several *E. sakazakii*-related outbreaks and sporadic cases, PIF was epidemiologically or microbiologically established as the source of infection [8-13]. This organism was however isolated at very low levels from commercial PIF and dry environmental samples collected from infant formula factories. The contamination was in most cases not exceeding 1 CFU per 100 g [14-20].

Prompted by the health risks associated with *E. sakazakii*, the International Standard Organisation (ISO) and the International Dairy Federation (IDF) have recently jointly adopted a Technical Specification (TS 22964), quoted as a Reviewed Method for IDF (RM 210), defining a method for the detection of *E. sakazakii *in PIF. The method is based on the selective enrichment procedure developed by Guillaume-Gentil *et al. *[18]. Isolation of the organism involves (i) a preenrichment in buffered peptone water (BPW), (ii) an enrichment in modified lauryl sulfate tryptose broth (mLST), and (iii) a plating on the chromogenic *Enterobacter sakazakii *Isolation Agar (ESIA). Identification include (i) observation of yellow pigmentation for suspected *E. sakazakii *colonies on tryptone soy agar (TSA) at 25°C and (ii) confirmation of the species via biochemical characteristics. The isolation and confirmation procedures usually require 5 to 7 days to be completed.

Although at present, the gold standard for the detection of microorganisms in food is in general conventional culture, molecular methodologies based on nucleic acid detection have been successfully developed these last years. They offer an interesting alternative to rapidly and specifically identify organisms from a wide variety of sources. With regards to *E. sakazakii *detection, several PCR-based approaches have already been published [21-26]. However, some of these methods do not include an internal amplification control (IAC) that allows a simultaneous assessment of PCR inhibition. Moreover, none of them have been evaluated with naturally contaminated food samples, thus not addressing the ecological diversity of naturally contaminated food matrices.

The present study evaluated a real-time PCR-based assay including an IAC and an automated nucleic acid extraction method that can be used in combination with the ISO-IDF enrichment steps for the routine examination of naturally contaminated PIF.

## Results

### Specificity of the real-time PCR assay

The DNA region located between the tRNA-glu and 23S rRNA genes was selected as target for detecting the *E. sakazakii *species. Primers ESFor and ESRevB were demonstrated to amplify a 158-bp fragment in all 35 strains of *E. sakazakii *tested with no cross-reaction with other non-*E. sakazakii *bacterial strains (data not shown). Real-time PCRs were next conducted with a first set of hybridisation probes in the presence of 450 molecules of IAC. All but one *E. sakazakii *strains gave a positive signal in real-time PCR assay (Table 1). For strain ES 10, we repeatedly failed to detect any fluorescent signal and this lack of *E. sakazakii *specific signal was curiously also accompanied by a lack of the IAC signal. To attempt to improve the assays, the ESFor/ESRevB amplified products specific to this strain were sequenced, as well as those of the strain ATCC 51329, one of the more phylogenetically distinct *E. sakazakii *strain which displayed one of the higher *C*_*T *_value in this assay (data not shown). Sequencing of both 158-bp target fragments did reveal some mismatches between the LC1ES hybridisation probes and its binding sequences. A new LC1ES probe incorporating three degenerated bases/positions compared to the initial one was therefore tested. The new designed real-time PCR was able to correctly detect all *E sakazakii *strains tested, including the ES 10 isolates. The use of a more degenerated LC1ES probe did not significantly affect the *C*_*T *_value obtained for each strain but increased the intensity of fluorescence measured for some of them, including strain ATCC 51329. This new probe was used in the rest of the study.

For the exclusivity test, a total of 139 non-*E. sakazakii Enterobacteriacea*e and 45 non-*Enterobacteriacea*e strains were chosen (Table 1). All 184 non-*E. sakazakii *strains tested were negative by either PCR or real-time PCR while a positive IAC signal was always detected (with a *C*_*T *_value above 37). The new *E. sakazakii*-IAC real-time PCR developed could therefore distinguish *E. sakazakii *isolates from non-target bacteria.

### Sensitivity of the real-time PCR assay

Two reference strains belonging to distinct lineages [21,27] were selected to determine the sensitivity of the assay. The type strain ATCC 29544 is representative of a majority of *E. sakazakii *strains. The previously mentioned strain ATCC 51329, the Preceptrol™ (quality control) strain, is more closely related to taxa including *E. pyrinus*, *E. hormaechei *and *C. koseri *[27].

**Figure 1:**
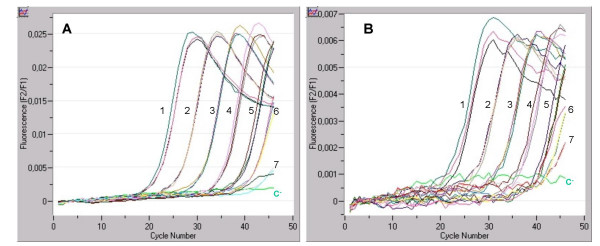
**Limit of detection of the real-time PCR determined from 10-fold serial dilution of genomic DNA**. Representative amplification plots obtained with 450 IAC molecules and decreasing amounts of *E. sakazakii *genomic DNA from *E. sakazakii *ATCC 29544 (A) and ATCC 51329 (B), equivalent to 10^6 ^(1), 10^5 ^(2), 10^4 ^(3), 10^3 ^(4), 10^2 ^(5), 25 (6), and 5 (7) copies per reaction. C^-^: negative control.

The sensitivity of the real-time PCR assay in the presence of 450 molecules of IAC was experimentally determined by using purified DNA. Tenfold dilutions of *E. sakazakii *genomic DNA in the concentration range of 10^6 ^to 10^0 ^equivalent genome per reaction were amplified in triplicate. Table 2 summarises the mean Ct values obtained for both strains. A detection limit of 5- and 25-genome copies was assessed for the strains ATCC 29544 and ATCC 51329, respectively. A positive reaction could however be detected in some cases with, respectively, as few as 1 and 5 DNA molecules per tube (Figure 1).

**Figure 2:**
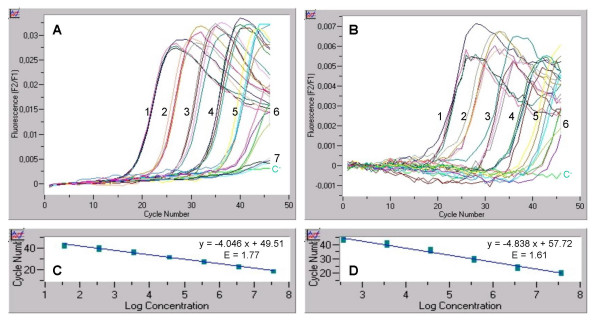
**Sensitivity of detection of the real-time PCR using pure culture of *E. sakazakii***. (A, B) Representative amplification plots obtained with 450 IAC molecules and decreasing concentration of cells of *E. sakazakii *ATCC 29544 (A) and ATCC 51329 (B): 3.6 × 10^7 ^(1), 3.6 × 10^6 ^(2), 3.6 × 10^5 ^(3), 3.6 × 10^4 ^(4), 3.6 × 10^3 ^(5), 3.6 × 10^2 ^(6) and 36 CFU/ml (7), corresponding to 1.8 × 10^6 ^(1), 1.8 × 10^5 ^(2), 1.8 × 10^4 ^(3), 1.8 × 10^3 ^(4), 1.8 × 10^2 ^(5), 18 (6) and 1.8 (7) cells per tube. C^-^: negative control. (C, D) Standard curves generated by plotting the log of cell numbers per tube of strain ATCC 29544 (C) or ATCC 51329 (D) versus the number of cycles required to reach the *C*_*T *_values.

To assess the detection limit of the assay combined with the DNA extraction step, tenfold serial cell dilutions of calibrated cultures containing 3.6 10^8 ^CFU/ml were performed and DNA extracted using the MagNA Pure LC system. Results showed that at least 36 and 360 CFU/ml (corresponding to 1.8 and 18 cells in the reaction tube) need to be present to get a positive reaction with the strains ATCC 29544 and ATCC 51329, respectively (Table 3). 100% of positive reactions were recorded in samples containing, respectively, 3.6 10^2 ^CFU/ml and 3.6 10^3 ^CFU/ml. Results showed a linear log correlation over the 10-fold dilution series (Figure 2), indicating that real-time PCR combined with MagNA Pure DNA extraction system could be used to quantify *E. sakazaki *cell suspensions.

### Evaluation of the real-time PCR assay coupled with the ISO-IDF enrichment procedure with artificially contaminated infant formula samples

Infant formula powders from three different commercial brands were inoculated with the strains ATCC 29544 or ATCC 51329 at four levels of contamination (1–5, 5–10, 10–20, 20–200 CFU/25 g) plus negative control. Briefly, twenty five grams of PIF were dissolved in 225 ml of buffered peptone water and then inoculated with diluted *E. sakazakii *culture. The contaminations were carried out in triplicate, except the blank which was twice repeated. Samples were analysed in parallel by the conventional ISO-IDF (TS 22964/RM 210) method and by real-time PCR after a common cultural enrichment. Both methods gave the same results for all samples and replicates (Table 4). Non-inoculated samples (blank) were negative. All but one artificially contaminated samples tested positive for *E. sakazakii*, demonstrating that both methods allow the detection of less than 5 *E. sakazakii *cells in 25 g of dried infant formulae. Mean *C*_*T *_values obtained by real-time PCR ranged from 19 to 26 cycles. As observed for the sensitivity analysis, *C*_*T *_values were systematically higher for the Preceptrol strain than for the *E. sakazakii *type strain ATCC 29544. A slight matrix-dependent effect was also noticed, with *C*_*T *_values for one of the infant formulae's brand higher than those of the others brands. In contrast, no significant difference was observed according to the contamination level, indicating that *E. sakazakii *has likely reached a similar growth density at the end of the enrichment procedure for all samples. The negative result found for one replicate by both methods suggested that the artificial contamination failed. Heterogeneity in artificial contamination at low inoculation levels (estimated to 4.5 cells) may explain the likely absence of *E. sakazakii *in this sample.

Given the reported presence of *E. sakazakii *at low level in PIF, a contamination level of 1 CFU/100 g was also attempted using the phylogenetically more distinct strain ATCC 51329 [21]. Out of the 5 replicates performed, 3 were positive for *E. sakazakii*. As previously mentioned, the 2 negative ones were likely not contaminated. The method is therefore able to detect as few as 0.01 cells per gram of dried infant formulae in as little as 48 h of total analytical time. In an attempt to further shorten this duration, we also tested whether the assay could be carried out after a single pre-enrichment of samples in BPW for 18 h. Equivalent results, with mean Ct values in the same range as previously found, were obtained (Table 4). The real-time PCR analytical time could therefore be shortened to about 24 h without reducing the method's sensitivity.

### Detection of ES in naturally contaminated samples: comparison of real-time PCR detection and conventional ISO-IDF culture method

The presence of *E. sakazakii *in a total of forty-one samples suspected to be naturally contaminated, including infant formulae and samples from the production environment of infant formulae factories, were investigated using the ISO cultural method and real-time PCR in parallel (Table 5). Twenty-five grams test portions were analysed in duplicate for each sample after BPW-mLST enrichments as recommended by the ISO-IDF procedure. Twenty-two samples were positive for *E. sakazakii *by the ISO-IDF method and 23 were tested positive by real-time PCR, giving more than 97.5% concordance between both methods. The sample (n° 21) that tested positive by PCR and negative by the culture method had a mean *C*_*T *_of 36.65. This value, largely higher than those found for the other positive samples (i.e. 19.15 to 26.82 cycles), indicated a lower *E. sakazakii *cell density in the enriched sample. Extrapolation of this value based on standard curves previously calculated (see Figure 2, Table 3) suggested that the cell load achieved at the end of the two-step enrichment might not exceed 10^3^–10^4 ^CFU/ml. Moreover, when tested after a single 18 h-BPW enrichment, *E. sakazakii *was not detected by real-time PCR in this sample in contrast to the other 22 positive ones (data not shown), indicating that growth was actually occurring during the second enrichment and that a detectable level could not be reached without a two-days incubation. All together, these data suggested that the additional PCR-positive sample was not a false positive result but an indication of the higher sensitivity of the real-time PCR assay compared to the conventional culture method.

## Discussion

Given the diversity and the limited sequence information available concerning the *E. sakazaki *genome, we selected the 16S–23S rDNA internal transcribed spacer (ITS) sequence to design a fluorescence resonance energy transfer (hybridisation-based) real-time PCR assay. This region has the advantages to be highly conserved throughout *Eubacteriae*, to somewhat differ in length and primary sequence between genus and species, and to be well characterised in numerous species and strains for phylogenetic purposes. The set of primers ESFor and ESRevB was shown to be specific enough to distinguish *E. sakazakii *from other isolates of bacteria by conventional PCR amplification. They amplified a 158-pb DNA fragment located between the tRNA-Glu and 23S rRNA genes. Taking account of the sequence variations encountered in this region among our collection of *E. sakazakii *isolates, the upstream internal probe LC1ES designed was degenerated in several positions to match the requirement for 100% homology between sequences of probes and templates. No false-positive or false-negative result was indeed obtained, indicating that the FRET real-time PCR assay developed was highly specific to *E. sakazakii*.

The diversity existing between *E. sakazakii *strains was found to affect the sensitivity of the real-time PCR results. *C*_*T *_values varied to some extent for same cell concentration, using different strains. While the detection limit was approximately 1 to 5 equivalent genome(s) per reaction for the strain ATCC 29544 (18 cells per PCR tube when combined with DNA extraction step), it was approximately 25 copies (180 cells per PCR tube when combined with DNA extraction step) for the phylogenetically more distinct strain ATCC 51329. The use of a very sensitive detection method is essential given the presence at low levels of *E. sakazakii *in PIF. An enrichment step was therefore included in the real-time PCR method to increase the bacterial population largely above the detection limit. The enrichment procedures recommended by the ISO-IDF (TS 22964/RM 210) method was evaluated for this purpose. This enrichment allowed to detect an initial contamination level of 1 cell per 100 g of PIF.

The PCR sensitivity depends on the efficiency of the method used to recover bacterial DNA from food. Sufficient nucleic acid extraction, with the removal of substances inhibitory to amplification, is important for an optimal detection of microbial pathogens by PCR, especially with a complex and rich food matrix such as PIF. The FRET real-time PCR was therefore combined with a robust nucleic acid extraction procedure, the MagNA Pure LC automated DNA extraction system. In this study, it was observed that by using the MagNA Pure system in combination with a LightCycler, reproducible results could be obtained with extraction of genomic DNA from PIF suspensions to real-time-PCR in approximately 2 to 3 h. Another interesting aspect of this system is the high level of purity of the resulting DNA. PCR detection in PIF is often hampered by PCR-inhibition problems (unpublished data). In this respect, the method offers a good DNA purification step prior to amplification and was shown to be satisfactory for analysis of PIF. Moreover, to assess PCR inhibition, a competitive IAC template was incorporated in the design of the assay. All real-time PCR reactions were performed in the presence of a given number of its copies. This allowed to distinguish negative responses due to the absence of target sequence in the sample from negative responses due to an amplification failure. All negative results in this study were then systematically validated by the IAC amplification.

Despite the use of an automated DNA extraction system, matrix-dependent effects affecting real-time PCR results were noticed. Mean *C*_*T *_values obtained from one of the three brands of artificially contaminated PIF used were found to differ from the others, being systematically slightly higher. The PIF composition is suspected to directly and significantly affect the PCR assay sensitivity. Compared to the 2 other brands, the involved PIF contained higher fat and skimmed milk content and was enriched with starch and *Bifidobacterium*. In addition, this powder was more difficult to handle (i.e., separating cells from the food suspension). The shift in *C*_*T *_values for this matrix might also reflect less favourable growth conditions which would not support the same cell density as the other PIF, linked perhaps to the presence of competitive microflora. However, the methodology developed, combining real-time PCR detection, automated DNA extraction and enrichment, was able to correctly identify all samples containing *E. sakazakii *irrespective of PIF brand, contamination level and strains analysed.

Reliable detection of pathogens in a timely manner is a major challenge for product quality control in the food industry, for official controls and for outbreak investigation. In this respect, an interesting strategy has been recently reported by Mullane *et al. *to quickly detect *E. sakazakii *in PIF [28]. The developed method requires a short enrichment period (6 h), followed by capture of the bacteria using charged paramagnetic beads and subsequent identification after plating onto selective agar. Real-time PCR technology coupled to automated nucleic acid extraction also meets this requirement. However, the method developed in this work includes a significant enrichment time that does not enable a same-day analysis. The enrichment procedure recommended by the ISO-IDF method that we used involves a pre-enrichment in BPW for 18 h, followed by an enrichment in mLST for 24 h. To shorten the total analytical time to less than 24 h, the enrichment step was limited to the single pre-enrichment in BPW. A clear real-time PCR positive signal was obtained with this one-step enrichment for all artificially or naturally contaminated samples, previously found positive using the two-step ISO-IDF enrichment, with one exception. Significant difference in term of *C*_*T*_, with a shift to more elevated values, were however observed with very low level of contamination, i.e., 1 CFU/100 g of PIF, and naturally contaminated samples, indicating a lower cell density at the time of the analysis for those samples. For one naturally contaminated sample (n° 21), *E. sakazakii *was only detectable by the assay with a two-step enrichment, due to probable late and laborious cell recovery. The presence in PIF and the environment of infant formulae factories of injured or stressed cells due to the type of process is plausible. In this case, an increased variability of individual lag times and of the detection times (i.e., the time by which a single-cell-generated subpopulation reaches a detectable level) is expected [29,30]. The second enrichment in mLST might therefore be useful for samples containing a low number of injured or stress cells. In this study, 1 of 41 samples was concerned. The analysis of more naturally contaminated samples need to be carried out to assess the frequency of such event and to assess whether the second enrichment might be omitted without the risk of false-negative results.

## Conclusion

Compared to the ISO-IDF cultural method, a high degree of agreement with the new developed real-time PCR method was found. Agreement (100%) was obtained with artificially contaminated PIF samples and 97.5% with naturally contaminated samples. The PCR detection system gave one additional positive. This result might suggest that, besides being significantly faster, molecular methods could also be more sensitive than the cultural method. In conclusion, the method developed can be considered as a fast alternative for identification of *E. sakazakii *in PIF.

## Methods

### Bacterial strains

The strains used in this study are listed in Table 1. Thirty-five *E. sakazakii *strains, whose identification was confirmed by biochemical test kit (ID32E, bioMérieux, Marcy l'Etoile, France), were used for selectivity testing. These included twenty-four strains isolated from infant formulaes factories and 5 strains isolated from dehydrated infant formulae. The *E. sakazakii *strains were grown aerobically at 37°C in BHI medium. The final BHI culture contained approximately 3.6 10^8 ^CFU/ml. A total of 139 non-*E. sakazakii Enterobacteriacea*e and 45 non-*Enterobacteriaceae *strains were selected for exclusivity testing (Table 1), with an emphasis on *Enterobacteriaceae *closely related to *E. sakazakii *such as *E. gergoviae*, *E. hormaechei*, *E. kobei*, *E. pyrinus*, *Enterobacter *sp. or *Citrobacter koseri *(Iversen *et al.*, 2004). Most of the strains were food and environmental isolates and were representative of food pathogens and other epidemiologically important species.

### Bacteriological method of reference

*E. sakazakii *detection procedure was performed according to the ISO-IDF method. Briefly, a non selective enrichment was performed at 37°C in buffered peptone water (BPW, AES Laboratoires, Combourg, France) for 18 h, followed by a selective enrichment in modified lauryl sulfate tryptose broth (mLST). One hundred microliters of the BPW suspension was transferred into 10 ml of mLST and further incubated at 44°C for 24 h. mLST contains per litre of deionised water: 35.6 g lauryl sulfate tryptose broth powder (AES Laboratoires), 29.2 g NaCl (Fisher scientific, Elancourt, France) and 10 mg vancomycin (Sigma, Steinheim, Germany), pH 6.8. The enrichment broth was then isolated on ESIA™ selective agar (AES Laboratoires). ESIA™ plates were incubated at 44°C for 24 h. Typical colonies (blue-green) were streaked on TSA (Oxoid, Dardilly, France) and incubated at ambient temperature (approximately 20°C) for 72 h to test the production of a yellow pigment. Presumptive colonies were lastly submitted to biochemical characterisation, using ID32E strips (bioMérieux, Marcy l'Etoile, France).

### Preparation of DNA samples

DNA purification from pure *E. sakazakii *cultures or from food suspensions was performed using the MagNA Pure LC DNA Isolation Kit III for bacteria and fungi on the MagNA Pure system (Roche Diagnostics, Meylan, France), according to the manufacturer's recommendations. Briefly, a 1-ml aliquot of the enriched culture was collected after centrifugation at 10000 × g for 3 min at 4°C. The supernatant was carefully discarded and the cell pellet was washed with 1 ml PBS (Phosphate Buffered Saline, Roche Diagnostics, France). After centrifugation, the cell pellet was resuspended in 100 μl of PBS and mixed with 130 μl of lysis buffer and 20 μl of proteinase K (Roche Diagnostics, France). The suspension was incubated successively at 65°C for 10 min and 95°C for 10 min and allowed to cool down before being transferred to the MagNA Pure system. DNA was eluted in 100 μl. A 2-μl aliquot of the eluted DNA was used as template for real-time PCR assay, except for DNA extracted from pure cultures which was 100-fold diluted in water before use.

DNA from pure non-*E. sakazakii *cultures was mostly extracted using a 200 μl aliquot of InstaGene™ Matrix (Bio-Rad Laboratories, Marnes-la-Coquette, France). Bacterial DNA was released by heating the sample for 20 min at 56°C and 10 min at 95°C. After vortexing and centrifugation (10000 × g, 4°C, 3 min), 2-μl of the supernatant was used for PCR assay.

DNA of plasmid T104 which contained the IAC construction used as internal amplification control was purified by using a QIAGEN plasmid midi kit (Qiagen, Courtaboeuf, France) according to the manufacturer's instructions.

All DNA preparations were stored at -20°C until use.

### Sequencing

Partial DNA sequences of the 16S–23S rDNA internal transcribed spacer (ITS) from *E. sakazakii *strains ES 10 and ATCC 51329 were determined. Primers ESFor and ESRevB were used to amplify the DNA from both strains using the Expand high fidelity PCR system (Roche Diagnostics). PCRs were carried out with a GeneAmp PCR system 9700 thermocycler (Applied Biosystems). A 50 μl PCR reaction contained 0.4 μM both primers, 200 μM each deoxynucleoside triphosphate (Roche diagnostics), 1 × PCR buffer without MgCl_2 _(Roche Diagnostics), 1.7 mM MgCl_2_, 2.6 U of High fidelity PCR DNA polymerase (Roche Diagnostics) and 1 μl of genomic DNA (approximately 30 ng). The reaction conditions were 94°C for 2 min followed by 35 cycles of 94°C for 30 s, 58°C for 30 s, and 72°C for 30 s. A final extension step of 72°C for 7 min was performed. The 158-bp amplicon generated was purified using the QIAquick purification kit (Qiagen) according to the manufacturer's instructions and sequenced by 'Genome-Express' (Meylan, France). The EMBL accession numbers for those sequences are AM295804 (strain ATCC 51329) and AM295805 (strain ES 10).

### Detection limit of the real-time PCR

The limit of detection of the LC-PCR assay was determined in the presence of a defined number of IAC copies, by using a serial dilution of purified genomic DNA and a serial dilution of a culture of known concentration. Ten ml of TBS (Oxoid, Dardilly, France) were inoculated with 100 μl of an overnight culture of the strain ATCC 51329 or ATCC 29544 and incubated for 6 h at 37°C to the late-exponential growth phase (approximately 4 × 10^8 ^CFU/ml). The cell suspension was next 10-fold serially diluted in buffered peptone water (Oxoid) to achieve a final concentration range of 10^8 ^to 10^1 ^CFU/ml. The exact number of CFU per millilitre was determined *a posteriori *by plating 100 μl of the 10^-5^, 10^-6 ^and 10^-7 ^dilutions onto TSA agar (Oxoid) in triplicate and by incubating the plates for 24 h at 37°C. The average number of CFU from six plates was used to estimate the concentration. Two 1-ml aliquots of each dilution were collected by centrifugation and the DNA was extracted from each cell concentrate and strain using the MagNA Pure system. A 5-μl aliquot of the eluted DNA was used as template. The real-time PCR amplification was carried out in the presence of 450 IAC copies as previously described. PCRs were performed in duplicate.

The genomic DNA purified from *E. sakazakii *ATCC 51329 or ATCC 29544 used as template was extracted from the non-diluted cell suspensions mentioned above. It was quantified by fluorimetry using PicoGreen (Molecular Probes, Eugene) in a TD-700 fluorimeter (Turner designs). The number of genomic copies of purified DNA was calculated as follows: *m *= *n *× (1.013 × 10^-21 ^g/bp), where *m *is the mass and *n *is the number of base pairs. The number of kilobase pairs for one *E. sakazakii *genome was assessed to be 4,500 Kb (according to the ATCC BAA-894 genome sequencing project performed at Washington University-GSC [31]). Consequently, one *E. sakazakii *genome weighs about 5.0 fg. Each DNA dilution in the concentration range of 10^6 ^to 1 genomic DNA copies per PCR (equivalent genome per PCR) was run in duplicate.

### Analysis of artificially and naturally contaminated PIF

Infant formulae from 3 different brands were locally purchased at retail, and used to assess the sensitivity in food matrices of the real-time PCR assay combined with *E. sakazakii *ISO-IDF enrichment procedures. Infant formula powders were artificially inoculated with individual *E. sakazakii *strain (strains ATCC 51329 and ATCC 29544) at four levels of contamination (1–5, 5–10, 10–20, 20–200 CFU/25 g), plus a negative control (blank), and submitted to the ISO-IDF detection protocol. Experiments were repeated in triplicate for all contamination levels, except the blank which was repeated twice. The exact numbers of *E. sazakakii *cells introduced into the food were determined *a posteriori *by plating 100 μl of 10^-5^, 10^-6 ^and 10^-7 ^dilutions on TSA agar in triplicate and by incubating the plates for 24 h at 37°C. The concentration was estimated by calculating the average number of CFU from at least six plates.

To give an outlook of the analytical procedure, 25 g of the spiked powdered formula samples were dissolved in 225 ml of BPW and incubated for 18 to 20 h at 37°C. One hundred microliters of the last suspension were subsequently inoculated into 10 ml of mLST and further incubated at 44°C for 24 h. Two 1-ml aliquots of the enrichment culture were taken and subjected to DNA extraction using the MagNA Pure Roche LC instrument.

For real-time PCR assays, DNA was extracted from 1-ml aliquots of either BPW or mLST enrichments in duplicate and all samples were analysed twice by real-time PCR as described above. Naturally contaminated samples were examined as described above. 25 g test portions were analysed in duplicate. All samples were kept closed at ambient temperature before use.

### Primers and probes used in real-time PCR

The design of primers and hybridisation probes was based on a multiple alignment of the intergenic 16S–23S rRNA sequences including *E. sakazakii *and other *Enterobacteriaceae*. The set of degenerated primers ESFor (5'ATCTCAAAAMTGACTGTAAAGTCACGTT3') and ESRevB (5'CCGAARAAGTMTTCGKGCTGCGA3') allowed to PCR-amplify an *E. sakazakii *specific target region of 158-bp located between the tRNA-Glu and 23S rRNA genes. The target probes are two oligonucleotides that are specific to the internal sequence of the amplified fragment. The upstream probe is labelled at the 3' end with fluorescein, whereas the downstream probe is labelled at the 5' end with 640RED fluorophore. The 3' end of the downstream probe was phosphorylated to prevent probe extension. During the annealing step of the amplification cycle, the two probes hybridised to the *E. sakazakii *template and were only two bases apart to allow FRET between the two fluorophores. Amplification of the target sequence is proportional to the fluorescence emitted by the 640RED dye monitored on the F2 channel of the Light-Cycler instrument. The internal FRET hybridisation probes have the following sequences: LC1ES (5'ACGGA(G/R)(A/R)AATRCA(G/R)CAGCRTGTCT3'-Fluo) and LC2ES (Red640-5'TTCAATTTTCAGCTTGTTCCGGATTGT3'-Phosphate). The specific probe used for IAC detection was LC150-5'Red705 (Red705-5'CTATCCTTGAGCCGTAGGCCACTATC3'-Phosphate). Generation of fluorescence linked to IAC amplification was monitored in channel F3 (705 nm). Primers and probes were purchased by Sigma-Proligo (France).

### Construction of an internal amplification control (IAC)

The IAC is a recombinant pMos*Blue *plasmid DNA (GE Healthcare Europe, Saclay, France) with ESFor and ESRevB primer binding regions, flanking a DNA sequence complementary to the LC1ES and LC150-5'Red705 probes. It was designed to be amplified in the same reaction as the *E. sakazakii *target region by using the same amplification primers ESFor and ESRevB. To construct this plasmid, a chimeric DNA fragment was generated by two runs of PCR. The first used the genomic DNA of strain ES1 as template and the set of primers ESCIA (5'TTAATATCTCAAAACTGACTGTAAAGTC3') and ESCIB (5'ACGGAGAAATACAGCAGCGTGTCTGTCTATCCTTGAGCCGTAGGCCACTATCTAAAGAGCAAATATCTCAAAC3'). The second PCR run used the purified first-round PCR product (thousand-fold diluted) as template and primers ESCIA and ESCID(5'ACAACCCGAAGAAGTCTTCGTGCTGCGAGTTTGAGAGACTCTGACACACCGCGCATTTCTTATTACGGAGAAATACAGCAGCGTGTCT3'). PCR conditions were 0.4 μM primers, 200 μM each deoxynucleoside triphosphate (Roche), 1 × PCR buffer without MgCl_2 _(Roche), 1.7 mM MgCl_2_, 2.6 U of High fidelity PCR DNA polymerase (Roche Diagnostics) and 1 μl of genomic DNA. The thermal profile consisted of an initial denaturation at 94°C for 2 min followed by 30 s at 94°C, 30 s at 50°C and 30 s at 72°C for 40 cycles, and a final elongation at 72°C for 7 min. The amplicon obtained was next purified with the QIAquick purification kit (Qiagen, Courtaboeuf, France) and cloned into *E. coli *MOS Blue by using a pMOSBlue blunt-ended cloning kit (GE Healthcare Europe, Saclay, France). The resulting plasmid T104 was quantified by fluorimetry using PicoGreen (Molecular Probes, Eugene) and a TD-700 fluorometer (Turner designs). The copies' number of the vector was calculated according to its size. For use in real-time PCR, the IAC plasmid was freshly diluted to the working concentration of 180 copies per μl in double-distilled water. The optimal concentration of IAC for incorporation into the PCR assay was determined empirically by adding different numbers of IAC copies to reactions containing serially diluted *E. sakazakii *genomic DNA. The goal was to determine the lowest reproducible IAC concentration that amplified consistently but did not interfere with the amplification of the target sequence.

### Real-time PCR conditions

Real-time PCR reactions performed with the LightCycler^® ^1.5 instrument (Roche Diagnostics) used a total volume of 20 μl, which was contained in a glass capillary tube. Optimisation experiments were first performed to choose the right IAC concentration and magnesium concentrations. The optimal amplification reaction mixture contained 1× LightCycler Faststart DNA master hybridization probes mix (Roche Diagnostics), 3 mM MgCl_2_, 500 nM of each primer (ESFor and ESRev), 200 nM of each probe (LCES1, LCES2 and LC150-5'Red705), 450 copies of plasmid T104 (IAC) and 1 to 2 μl of the tester DNA. Thermal cycling was carried out by using an initial denaturation step at 95°C for 10 min, followed by 46 cycles of denaturation at 95°C for 10 s, annealing at 60°C for 10 s and extension at 72°C for 15 s, with a temperature transition rate of 20°C/s except for the annealing step for which it was 2°C/s. Cycling was completed by a final cooling step at 40°C for 30 s. Generation of fluorescence for each sample was recorded at the 60°C annealing step in channel F2/F1 (640 nm) for *E. sakazakii *target DNA amplification and in channel F3/F1 (705 nm) for IAC amplification. Each real-time PCR assay systematically included three control reactions performed in parallel to the tested samples. These controls used as template a hundred-fold diluted purified genomic DNA of *E. sakazaki *strain ES 1, water, or 450 copies of IAC, respectively

### Data analysis

The cut-off values (background fluorescence baseline) were set above the highest end-point fluorescence signals of the negative controls. Samples with threshold cycle values (*C*_*T*_) of less than 44 cycles were considered positive. The *C*_*T *_value indicates the cycle at which sample fluorescence signal is first recorded as statistically significant above background fluorescence. Usually it occurs when the signal detection software begins to detect the increase in signal associated with an exponential formation of PCR product. The more template is present at the beginning of the reaction, the fewer cycles it takes to reach this point.

## Authors' contributions

FD and SD carried out the PCR experiments. SD designed the assay and drafted the manuscript. All authors read and approved the final manuscript.

## Figures and Tables

**Table 1:** Bacterial panel used to evaluate the specificity and sensitivity of the real-time PCR assay

**Species/Serotypes**	**nb**	**Strains (source)**	**Species/Serotypes**	**nb**	**Strains (source)**
*Enterobacter sakazakii*	35	ATCC 29544 (CIP)	*Salmonella *Hadar	2	(AFSSA)
		CIP 104951 (CIP)	*Salmonella *Typhimurium	2	(AFSSA, BgVV)
		CIP 104952 (CIP)	*Salmonella *Typhi	1	(AFSSA)
		ES 1, ES 2, ES 5,	*Salmonella *Kottbuss	1	(AFSSA)
		ES 9, ES 10, ES 16,	*Salmonella *Virchow	1	(AFSSA)
		ES 17, ES 18, ES 19,	*Salmonella *Derby	1	(AFSSA)
		ES 20, ES 23, ES 28,	*Salmonella *Enteritidis	2	(AFSSA, BgVV)
		ES 29, ES 30, ES 32,	*Salmonella *Bredeney	1	(BgVV)
		ES 34, ES 37, ES 40,	*Salmonella *Agona	1	(AFSSA)
		ES 41, ES 44, ES 52,	*Salmonella *Indiana	1	(AFSSA)
		ES 53 (IFF)	*Salmonella *Saintpaul	1	(AFSSA)
		ES8110043 (BM)	*Salmonella *Montevideo	1	(AFSSA)
		ES8311015 (BM)	*Salmonella *Anatum	1	(AFSSA)
		ES8704047 (BM)	*Salmonella *Dublin	1	(BgVV)
		ATCC 51329 (BM)	*Salmonella *(II)	1	(BgVV)
		ES8405628 (BM)	*Salmonella *Senftenberg	1	(AFSSA)
		ES810726 (BM)	*Salmonella *(IIIa)	1	(AFSSA)
		HMPL118 (AFSSA)	*Salmonella *(IIIb)	1	(BgVV)
		HMPL194 (AFSSA)	*Salmonella *Arizonae	1	(AFSSA)
		HMPL1937 (AFSSA)	*Serratia fonticola*	1	CIP 78.64 (CIP)
		HMPL1740 (AFSSA)	*Serratia liquefaciens*	4	(CTSCCV, AFSSA)
*Enterobacter gergoviae*	1	ATCC 33028 (CIP)	*Serratia marcescens*	3	(AFSSA)
*Enterobacter hormaechei*	1	ATCC 49162 (CIP)	*Raoultella planticola*	1	CIP 81.36 (CIP)
*Enterobacter kobei*	1	ATCC BAA-260 (CIP)	*Yersinia enterocolitica*	2	(AFSSA)
*Enterobacter pyrinus*	1	ATCC 49851 (CIP)	*Aeromonas putrefaciens*	1	(AFSSA)
*Enterobacter sp.*	1	CIP 53.120 (CIP)	*Aeromonas hydrophila*	1	(AFSSA)
*Enterobacter amnigenus*	4	(CTSCCV, AFSSA)	*Acinetobacter sp.*	1	(AFSSA)
*Enterobacter cloacae*	18	(AFSSA)	*Acinetobacter baumanii*	1	CIP 70.34 (CIP)
*Enterobacter aerogenes*	5	(AFSSA)	*Acinetobacter johnsonii*	1	(AFSSA)
*Enterobacter agglomerans*	4	(AFSSA)	*Acinetobacter lwoffii*	1	CIP 104272 (CIP)
*Enterobacter intermedius*	3	(AFSSA)	*Pseudomonas fluorescens*	2	(AFSSA)
*Citrobacter freundii*	^8^	(AFSSA)	*Pseudomonas aeruginosa*	2	(AFSSA)
*Citrobacter koseri*	6	i.e. ATCC 27028 (CIP, AFSSA)	*Stenotrophomonas maltophilia*	1	(AFSSA)
*Citrobacter sp.*	1	(AFSSA)	*Campylobacter jejuni*	2	CCUG 11284
*Citrobacter farmeri*	1	(AFSSA)	*Alcaligenes tolerans*	1	CIP55.94 (CIP)
*Escherichia coli * (i.e. O86; O127:H6; O157:H7; O55; O145; O113:H2; O103)	12	i.e. NCTC12900 (AFSSA)	*Alcaligenes faecalis*	1	CIP62.32 (CIP)
			*Listeria monocytogenes*	5	i.e. clip 74902 (CIP, AFSSA)
*Escherichia vulneris*	1	(AFSSA)	*Listeria innocua*	1	(AFSSA)
*Escherichia hermanii*	1	(AFSSA)	*Listeria weshimeri*	1	ATCC 35897
*Escherichia fergusonnii*	1	(AFSSA)	*Listeria seeligeri*	1	Clip 12513 (CIP)
*Buttiauxella agrestis*	1	(AFSSA)	*Listeria ivanovii*	1	Clip 74915 (CIP)
*Hafnia alvei*	5	(AFSSA)	*Enterococcus faecalis*	2	(AFSSA)
*Klebsiella pneumoniae*	8	ATCC 13883	*Enterococcus faecium*	1	(AFSSA)
*Klebsiella terrigena*	1	(CTSCCV)	*Bacillus cereus*	4	(AFSSA)
*Klebsiella oxytoca*	3	(AFSSA)	*Bacillus subtilis*	1	CIP52.65 (CIP)
*Morganella. morganii*	1	(AFSSA)	*Bacillus megaterium*	1	(AFSSA)
*Pantoea sp.*	3	(AFSSA)	*Bacillus lentus*	1	(AFSSA)
*Pantoea agglomerans*	1	ATCC 27155	*Staphylococcus aureus*	3	i.e. ATCC 700699
*Providencia alcalifaciens*	1	(AFSSA)	*Staphylococcus haemolyticus*	1	CIP 81.56 (CIP)
*Providencia stuartii*	1	(AFSSA)	*Staphylococcus simulans*	1	CIP 81.64 (CIP)
*Proteus vulgaris*	2	(AFSSA)	*Staphylococcus lentus*	1	CIP 81.63 (CIP)
*Proteus morganii*	3	(AFSSA)	*Staphylococcus equorum*	1	CIP 103502 (CIP)
*Proteus mirabilis*	2	(AFSSA)	*Staphylococcus capitis*	1	CIP 81.53 (CIP)
*Photorhabdus luminescens*	1	TTO1 (CIP)	*Streptococcus dysgalactiae*	1	CIP 102914 (CIP)
*Shigella sonnei*	2	(AFSSA)	*Streptococcus equinus*	1	CIP 103232 (CIP)
*Shigella flexneri*	1	ATCC 12022	*Streptococcus bovis*	1	CIP 102302 (CIP)

IFF : 22 isolates from infant formulae factories, kindly provided by A. Leclercq (Institut Pasteur, Paris, France); BM : bioMérieux (Craponne, France) kindly provided by I. Desforges; BgVV: Bundesinstitut für gesundheitlichen Verbraucherschutz und Veterinärmedizin (Germany); CTSCCV: Centre Technique de la Salaison, de la Charcuterie et des Conserves de Viandes (Maisons Alfort, France); CIP : Culture Collection of Institut Pasteur (Paris, France); AFSSA : Agence Française de Sécurité Sanitaire des Aliments (Maisons-Alfort, France).

**Table 2:** Sensitivity of real-time PCR for detection of a serial 10-fold diluted *E. sakazakii *DNA in presence of IAC

	Strain 29544		Strain ATCC 51329	
	
Nb. of genome copies/PCR	*C*_*T *_value (target)	*C*_*T *_value (IAC)	*C*_*T *_value (target)	*C*_*T *_value (IAC)
10^6^	19.60 ± 0.15	-	23.79 ± 0.23	-
10^5^	23.75 ± 0.16	-	28.24 ± 0.50	-
10^4^	27.99 ± 0.98	-	32.11 ± 0.49	-
10^3^	32.86 ± 0.03	> 38	37.04 ± 0.71	39.44 ± 1.20
10^2^	36.27 ± 0.68	40.85 ± 1.11	40.56 ± 0.86	41.10 ± 0.99
50	37.67 ± 0.21	40.53 ± 0.85	41.49 ± 0.75	40.32 ± 0.22
25	38.42 ± 0.46	40.24 ± 1.53	42.09 ± 0.97	41.20 ± 0.62
10	38.99 ± 1.82	37.14 ± 1.66	43.73 ± 1.41	41.29 ± 1.84
5	40.79 ± 1.41	38.77 ± 2.95	> 43.7	40.22 ± 1.77
1	> 44	40.46 ± 0.87	> 45	39.86 ± 0.27

*C*_*T *_values are means ± standard deviation obtained for three replicates

**Table 3:** Sensitivity of real-time PCR for detection of *E. sakazakii *in broth culture

*E. sakazakii *concentration		Strain ATCC 29544		Strain ATCC 51329	
CFU/ml	CFU/PCR	*C*_*T *_value (target)	*C*_*T *_value (IAC)	*C*_*T *_value	*C*_*T *_value (IAC)
3.6 10^7^	1.8 10^6^	18.52 ± 0.07	-	21.90 ± 0.43	-
3.6 10^6^	1.8 10^5^	22.86 ± 0.30	-	26.46 ± 0.56	-
3.6 10^5^	1.8 10^4^	27.49 ± 0.32	-	31.83 ± 0.62	-
3.6 10^4^	1.8 10^3^	31.76 ± 0.15	-	37.12 ± 0.46	> 42
3.6 10^3^	1.8 10^2^	36.19 ± 0.39	37.90 ± 1.51	41.89 ± 1.01	40.51 ± 0.97
3.6 10^2^	18	39.55 ± 1.15	38.12 ± 1.16	> 44.85	39.08 ± 1.62
36	1.8	42.46 ± 0.54	38.10 ± 1.92	-	39.36 ± 1.10

*C*_*T *_values are means ± standard deviation obtained for two independent DNA extractions with two amplification repetitions per extraction

**Table 4:** Detection of *E. sakazakii *in inoculated infant formula samples

	ATCC 29544				ATCC 51329			
	
		ISO	real-time PCR (C_*T *_values)			ISO	real-time PCR (C_*T *_values)	
Powdered Infant Formula	Seeding level (CFU/25 g)		BPW	BPW-mLST	Seeding level (CFU/25 g)		BPW	BPW-mLST
	0	- -	- -	- -	0	- -	- -	- -
	4	+++	17.68 ± 1.27	19.24 ± 0.26	4.5	+++	25.75 ± 3.44	23.70 ± 2.09
PIF 1	8.8	+++	18.95 ± 1.53	19.48 ± 0.20	9.5	+++	23.40 ± 0.93	24.15 ± 0.72
	18	+++	20.46 ± 3.36	18.99 ± 0.46	19	+++	20.78 ± 2.11	22.61 ± 0.27
	100	+++	nd	19.25 ± 0.39	103.5	+++	21.33 ± 0.57	23.51 ± 1.73

	0	- -	- -	- -	0	- -	- -	- -
	4	+++	nd	22.26 ± 0.42	4.5	+ - +	33.40 ± 4.14	24.53 ± 1.13*
PIF 2	8.8	+++	nd	21.73 ± 0.39	9.5	+++	31.99 ± 5.85	25.31 ± 1.83
	18	+++	nd	21.22 ± 0.66	19	+++	33.46 ± 1.86	26.44 ± 0.62
	100	+++	20.27 ± 3.77	21.31 ± 0.53	103.5	+++	32.95 ± 1.19	25.26 ± 2.66

	0	- -	- -	- -	0	- -	- -	- -
	0.25	nd	nd	nd	0.25	nd	24.74 ± 0.41*	22.75 ± 0.32*
	0.375	nd	nd	nd	0.375	nd	27.40 ± 0.45^§^	21.90 ± 0.02^§^
PIF 3	4	+++	16.39 ± 0.66	19.69 ± 1.12	4.5	+++	21.72 ± 0.24	24.43 ± 3.03
	8.8	+++	19.51 ± 2.44	19.40 ± 0.40	9.5	+++	21.80 ± 1.61	22.16 ± 1.22
	18	+++	18.73 ± 0.49	19.42 ± 0.57	19	+++	21.59 ± 0.96	22.12 ± 1.03
	100	+++	18.89 ± 0.32	19.29 ± 1.80	103.5	+++	20.67 ± 0.41	21.71 ± 0.46

*C*_*T *_values are means ± standard deviation for 3 independent experiments. +: detection of *E. sakazakii*; -: no detection of *E. sakazakii*; nd: not done; *C_*T *_value of 2 out 3 independent experiments analysed in duplicate; ^§^*C*_*T *_value of 1 out 2 experiment analysed in duplicate.

**Table 5:** Comparison between ISO-IDF and real-time PCR methods on collected naturally contaminated samples

		real-time PCR (*C*_*T *_values)				real-time PCR (*C*_*T *_values)	
					
Sample number	ISO	1	2	Sample number	ISO	1	2
1	-	-	-	22	+	19.83	21.84
2	-	-	-	23	+	24.32	25.83
3	-	-	-	24	-	-	-
4	-	-	-	25	+	20.27	20.38
5	+	21	20.94	26	-	-	-
6	+	24.41	22.42	27	+	20.93	20.35
7	+	20.82	19.63	28	-	-	-
8	-	-	-	29	-	-	-
9	-	-	-	30	-	-	-
10	+	21.6	21.39	31	+	22.00	19.76
11	+	19.7	19.69	32	+	24.67	23.80
12	-	-	-	33	+	19.73	20.93
13	+	18.65	19.65	34	-	-	-
14	+	26.07	27.57	35	+	24.41	24.84
15	-	-	-	36	+	19.03	20.46
16	-	-	-	37	+	24.30	24.34
17	-	-	-	38	-	-	-
18	+	19.75	21.50	39	+	23.63	22.94
19	+	22.58	23.67	40	-	-	-
20	+	21.58	21.89	41	+	19.87	20.14
21	-	36.95	36.35				

+: detection of *E. sakazakii*; -: no detection
